# Nursing assistants: “He seems to be ill” – a reason for nurses to take action: validation of the Early Detection Scale of Infection (EDIS)

**DOI:** 10.1186/s12877-015-0114-0

**Published:** 2015-10-12

**Authors:** P. Tingström, A. Milberg, N. Rodhe, J. Ernerud, E. Grodzinsky, M. Sund-Levander

**Affiliations:** Department of Medical and Health Sciences, Linköping University, 58183 Linköping, Sweden; Palliative Education & Research Centre and Department of Social and Welfare Studies, Linköping University, 601 74 Norrköping, Sweden; Department of Public Health and Caring Sciences, Faculty of Family Medicine and Preventive Medicine, Uppsala University, 751 05 Uppsala, Sweden; Department of Clinical and Experimental Medicine, Linköping, Sweden; Department of Clinical Immunology and Transfusion Medicine, Linköping University, 58183 Linköping, Sweden; Department of Pharmaceutical Research, Linköping, Sweden

**Keywords:** Nursing home residents, Clinical assessment, Instrument validation, Instrument construction

## Abstract

**Background:**

Signs and symptoms of infection in frail elderly are atypical, causing delay in diagnosis and treatment. To improve communication between healthcare staff of signs and symptoms of infection we developed an instrument, using qualitative data from observations by nursing assistants when they suspected infection. The aim of this study was to assess the validity of nursing assistants observations by developing and testing the instrument for early detection of infection in elderly nursing home residents.

**Methods:**

The early detection of infection (EDIS) instrument was based on data from focus interviews with nursing assistants. Over one year the nursing assistants used EDIS to document episodes of suspected early signs and symptoms of infection in 204 nursing home residents. Two physicians classified documented episodes as “no infection”, “possible infection”, and “infection”. The content validity of the 13 items of the EDIS was established to explore the relationships between the items. The construct validity was used to explore the relationship between the items and the presence or absence of infection. The predictive value of the developed model was evaluated by the percentage of correct classifications of the observed cases. Generalized linear model (ordinal multinomial distribution and logit link) was used.

**Results:**

Of the 388 events of suspected infection, 20 % were assessed as no infection, 31 % as possible infection and 49 % as infection. Content validity analysis showed that 12/13 of the items correlated significantly with at least one other statement. The range in number of significant inter-correlations was from 0 (“pain”) to 8 (“general signs and symptoms of illness”). The construct validity showed that the items “temperature” , “respiratory symptoms” and “general signs and symptoms of illness” were significantly related to “infection”, and these were also selected in the model-building. These items predicted correct alternative responses in 61 % of the cases.

**Conclusion:**

The validation of EDIS suggests that the observation of “general signs and symptoms of illness”, made by nursing assistants should be taken seriously in detecting early infection in frail elderly. Also, the statement “He/She is not as usual” should lead to follow-up.

**Electronic supplementary material:**

The online version of this article (doi:10.1186/s12877-015-0114-0) contains supplementary material, which is available to authorized users.

## Background

Nursing home residents (NHR) are more likely than elderly in general to suffer from acute infections due to general frailty and physical incapability [1, 2]. Infectious diseases are associated with poorer prognosis, delirium [3, 4], hospital care and increased mortality [5, 6], and also with long rehabilitation and decreased physical function, which may also reduce general wellbeing. The prevalence of infections in NHRs is estimated to be 11 to 13 % [7, 8]. The most frequent are urinary tract infection (UTI) (5.2 %), pneumonia (2.2 %) and cellulitis (1.6 %), while other infections constitute 2.8 % of the total [8–10]. Cham et al. estimated pneumonia to account for 41 % of all infections [7]. In an earlier study we found that pneumonia was as common as stroke and heart failure as a cause of death in NHRs [11]. Signs and symptoms of infection in the frail elderly are often atypical, while specific ones, including fever, are often absent [9], causing a delay in diagnosis and treatment. Examples of atypical signs and symptoms are weakness, falling, weight loss, physical dysfunction and cognitive decline [9, 12–14]. In pneumonia, the presence of cognitive decline is as common as symptoms more specific to respiratory tract infection, such as cough and sputum production [14]. However, quite frequently clinical identification of infections among elderly is based on atypical symptoms and end up not being infections. For example, D’Agata et al. reported that mental status changes, such as lethargy or alterations of cognitive status, were the sole symptoms or signs documented in 36 % of episodes of suspected UTI in NHRs, and only 21 (16 %) of the 131 episodes met the minimum criteria to initiate antimicrobial therapy based on documented signs or symptoms [15]. Consequently, there is a risk of over treating elderly persons with antibiotics, and e.g. Sundvall et. al [16] reported that residence in a care home setting is associated with high antibiotic consumption, especially for UTI where the odds of prescription is doubled.

In nursing homes (NHs), the registered nurse (RN) is responsible for the assessment of the residents’ conditions. These assessments are often based on the subjective observations of nursing assistants (NA), who mostly communicate their observations to medical staff informally [17] and do not participate further in the decision-making process [7]. Although research has stressed the significance of atypical signs and symptoms in diagnosing infection in the elderly, the RNs and physicians seem to be more interested in typical medical signs and symptoms in the NAs’ reports than descriptions of changed behaviour [18]. Hence, in clinical practice the responsible RN and/or doctor may not take further action despite being in possession of important information about suspected infections, reported by NAs. To further improve identification of infections in NHRs, it seems important to take advantage of NAs’ observations of early signs and symptoms of infection since they provide most of the daily individual care of the NHRs [18, 19].

One way to improve the early identification of infection in NHRs is to improve the communication between the NA and the RN by using an evidence-based and standardized instrument of possible signs and symptoms exhibited by NHRs. Boockvar et al. [17] developed an illness warning instrument for short-term acute illness. However, this instrument did not specifically address signs and symptoms of suspected infection. In a previous qualitative study we have reported that NAs express changes that they relate to early non-specific signs and symptoms of infection in frail elderly in two categories: “He/She is not as usual” and “He/She seems to be ill”. The first category describes behavioural changes and discomfort, such as expression in the eyes, confusion, aggressiveness, infirmity/apathy, unrestrained, restlessness, and changed food intake, while the second more distinctly relates to well established specific signs and symptoms of infection, such as fever, shaking, shivering, paleness, flushed face [18]. A vital question is the credibility of the NAs’ observations, i. e. how valid are these observations for early detection of ongoing infection. To improve the communication of signs and symptoms of suspected infection, typical as well as atypical ones, to the RN we wanted to develop an instrument, the Early Detection of Infection Scale (EDIS), using qualitative data that NAs include in their reports when they suspect infection [18].

The aim of this study was to assess the validity of NAs’ observations by developing and testing the EDIS instrument for early detection of infection in elderly NHRs.

## Method

**Fig. 1:**
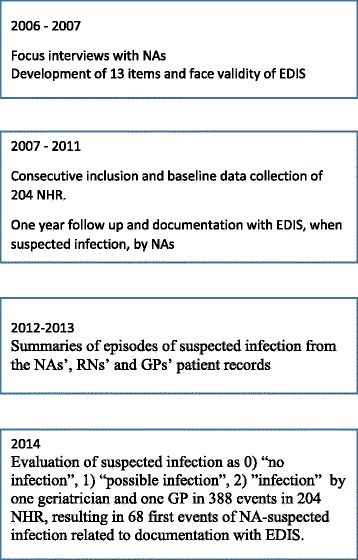
Flow-chart over the development of the Early Detection of Infection Scale (EDIS) instrument

In order to develop and validate the EDIS instrument, a cohort of NHRs was followed prospectively for one year (Fig. 1).

## NHR sample

The NHR sample consisted of 204 individuals, aged 66 to 101 years, mean age 86 years from 6 municipal non-profit long-term NHs in South of Sweden. Of these 204 individuals, 156 experienced 388 events, ranging from 1 to 8, of suspected infection, resulting in 288 antibiotic cures. In 165 (43 %) of the 388 events of suspected infection, the NAs documented the event by using EDIS. Only the first event of suspected infection was used in the development of the EDIS instrument. As a result, 68 individuals/events were included in this study. Background data are presented in Table 1.

**Table 1:** Background data in 68 elderly nursing-home residents, assessed for signs and symptoms of infection with the Early Detection of Infection Scale (EDIS)

Variable	*n* = 68
Age in years	84.5 ± 7.5
Male/female	23/45
BMI^a^	27 ± 4
ADL status^b^	8 ± 2
Ear temperature^c^	36,3 ± 0,4 °C
Rectal temperature	36,9 ± 0,3 °C
	n (%)
≥ 3 diagnosis	18 (26)
Dementia	49 (72)
Cognitive decline (MMSE^d^ 0–19)	20 (29)
Cardio-vascular disease	41 (60)
Chronic obstructive pulmonary disease	5 (7)
Stroke	25 (37)
Diabetes	16 (24)
Cancer	13 (19)
Autoimmune disease^e^	8 (12)
Thyroid disease	21 (10)
Cortisone ≥ 5 mg per dag	4 (6)
Sedatives/tranquillizers	28 (41)
Sleeping pills	20 (29)
Anti-depressants	39 (57)
Paracetamol ≥ 3 g daily	22 (32)
Malnutrition	6 (9)
Pain (DOLOPLUS ≥ 5)	44 (65)
Vaccination influenza	55 (81)
Vaccination pneumocockiae	31 (46)

The data are expressed as mean ± SD (age, BMI and ADL) or number of individuals

^a^Body mass index

^b^Activities of daily living

^c^Mean values and standard deviation was equal for ear temperature, irrespective if the left or right ear was used for measurement

^d^Mini mental state examination

^e^e.g. rheumatoid arthritis, muscular rheumatism

## Baseline measurements and data collection

A project nurse in cooperation with one researcher (MSL) collected all background information about the NHRs. Data on chronic diseases and medication were collected from medical records. The diagnosis of dementia was established according to ICD-10, and documented in medical records. Activities of daily living (ADL) were divided into personal (P)-ADL, consisting of the categories bathing, dressing, toileting, transfer, continence and feeding, and I-ADL, including cooking, transportation, shopping and cleaning. The residents were graded from 0 to 10, where 0 = independency, and 10 = dependency in all activities. Each dependency adds 1 point. Grade 5 means that the resident cannot manage any activities of I-ADL but can manage one P-ADL activity [20, 21]. Mini Nutritional Assessment (MNA) was used to assess nutritional status and BMI, i.e., kg/m^2^, was calculated [22]. Cognitive decline was assessed with the Mini Mental State Examination (MMSE) [23]. Pain was assessed with Doloplus-2, rating somatic, psychomotor and psychosocial behavioural changes as indicators of pain on a four-grade scale. On this scale, a total score of five or more is interpreted as the presence of pain [24, 25]. The presence of psychiatric signs and symptoms was assessed with the Neuropsychiatric Inventory - Nursing Home version (NPI-NH) on a 5-grade scale (0 = never – 4 = very often). Body temperature was measured in the ear with a Genius-2 (Covidien Sweden AB) or rectally (MC-638 Omron Health Care Europe BV, Täby, Sweden). Dipstick urine analysis for erythrocytes, leucocyte esterase and nitrites, was read analysed at the NH and C-reactive protein, white blood cells (CRP, WBC) and urine culture were analysed with accredited routine methods at Ryhov Hospital in Jönköping, Sweden.

## Procedure for development of EDIS

The development of EDIS proceeded in five steps.

1. Decisions about items, scale, method of data collection and presentation of results.
  As NAs communicate their observations to medical staff mostly informally [17], we decided to collect data in a systematic, structured written protocol. The 13 items, i.e. the EDIS instrument (see Additional file 1), were constructed from the two main categories, based on the results of the qualitative study of reported observations from NA [18]. The first main category named “He/She is not as usual”, included the subcategories i) expression in the eyes; ii) confusion; iii) aggressiveness; iv) infirmity/apathy; v) unrestrained behaviour; vi) restlessness; and vii) food intake. The second category named “he/she seems to be ill” contained viii) “general signs and symptoms of illness” and ix) “pain”, or more specific signs and symptoms of x) UTI; xi) respiratory infection; and xii) wound infection. The categories were constructed as items starting with the statement “there is a change in”. The instruction was to describe what was different compared to habitual condition, i e. a change could be the observed presence of a new symptom or a worsening of a previously existing symptoms. When the NAs, based on their observations, suspected infection they scored the items in EDIS in three levels: (0) do not agree; 1) partly agree; 2) completely agree. In addition, there was also a 13th item, asking for information on the patient’s body temperature (ear or rectal). The intention was to validate the relationship between the single item and suspected infection, and not to summarise the scores of EDIS.
2. Face validity
  The face validity was established in four steps. Firstly, two of the researchers (M S-L and PT) developed the draft of EDIS. Secondly, the instrument was presented to all the NAs who participated in the focus group interviews [18], and they judged it as valid - they had no further changes to suggest and that it was readable, understandable and complete. Thirdly, the draft was discussed in the research group and minor changes were made. Finally, the draft was presented and discussed with another group of five NAs by one of the researchers (M S-L) and a project nurse, resulting in no further corrections.
3. Data collection
  Before data collection started, the study and the preliminary EDIS were presented in written and oral forms by MS-L at staff meetings at the included NH. During the one-year period of follow-up of each participant the NAs were instructed to use the preliminary EDIS to document what they considered to be early signs and symptoms of suspected infection and to contact the RN. That is, all EDIS documentation was completed by the NAs before infection was identified by the RN or GP. During the follow-up, MS-L and the project nurse were in regular, weekly, contact with the NAs in order to support the use of EDIS and the data collection process. After the follow-up, MS-L and the project nurse read all journal records and compiled the documentation from the NA, RN and general practitioner (GP) patient records, making summaries of each episode of suspected infection by either the NA, RN and/or GP (*n* = 388). The summaries included NA’s EDIS documentation, and medical and nursing care records. Data on background factors was also added to each summary.’
  Finally, two experienced physicians, one geriatrician (AM) and one GP (NR) independently, based on the summaries, evaluated and classified each documented episode of suspected infection as 0) “no infection”, 1) “possible infection”, 2) ”infection”. Inter-reliability between the two physicians was tested in a pilot evaluation with 20 selected NHRs. The sample was selected by MS-L in order to reveal individuals with few as well as several episodes of suspected infection, with or without cognitive decline, and from different NHs. This pilot evaluation of episodes (*n* = 62 in 20 NHRs) where an NA had suspected infection resulted in 95 % full agreement (59/62) in the two physicians’ scoring. In the remaining cases (*n* = 3) consensus was achieved after discussion. The outcome of the pilot test confirmed the interrater reliability of the two evaluators.
4. User acceptance
  During the study period the NAs were repeatedly asked by the project nurse and MS-L about how they experienced the data collection and the preliminary EDIS instrument. The NA found the preliminary EDIS instructive and had no difficulty in understanding or scoring the statements. The scoring took approximately 1–2 min.
5. Statistical tests
  Descriptive statistics (mean + SD) were calculated for individual variables. Spearman Rank Order Bivariate correlations of the 13 items of the preliminary EDIS were performed to explore the relationships between the items, and the internal content (content validity) covered by the preliminary scale. The relationship between the items in the preliminary EDIS and the assessment by the two independent physicians of whether infection was present or not (construct validity), and the selected model was evaluated by Wald values derived from a generalized linear model analysis (GLZ), with an ordinal multinomial distribution and a logit link. The outcome variable in the model was “MD assessment of suspected infection”, defined as no infection (0), possible infection (1) or infection (2). The response variables considered for entry in the GLZ analyses were treated as continuous. In further model-building analyses a selection of identified variables from the univariate GLZ-analyses predicting infection in the NHRs were used. Classification of the developed model was carried out and the percentage of correct classifications of the observed cases was calculated. Sensitivity and specificity of the dichotomous predictive items in the model-building were calculated. Statistical significance was set as *p* < 0.05. The data were analysed using Statistica, v. 10 (Statsoft Inc., Tulsa, OK) and SPSS v 21(IBM Cooperation). In cases of missing values, the specific analysis was performed without this respondent, although the respondent was included in other analyses.

## Ethics

The study was conducted in accordance with the Declaration of Helsinki and was approved by The Ethics Committee for Human Research at the Faculty of Health Sciences, Linköping University (M82-06). Written informed consent from residents or next of kin was obtained after oral and written information given by M S L or the project nurse.

## Results

Of the 388 events of suspected infection in the total sample of 204 NHRs, 68 were first events of-suspected infections. Eighty of the 388 events (20 %) were assessed by the researchers (geriatrician and GP) as “no infection”, 123 (31 %) as “possible infection” and 195 (49 %) as “infection”. Of the 68 first events of suspected infections included NHRs in the testing of EDIS, the figures for suspected infection were 17 (25 %) for “no infection”, 16 (23 %) for “possible infection”, and 35 (51 %) for “infection”. When comparing background data, the 68 NHRs (included in the validation of EDIS) were more dependent in ADL (8 ± 2 vs 7 ± 2, *p* < 0.01), had a higher body mass index (BMI) (27 ± 4 kg/m^2^ versus 25 ± 5, *p* < 0.01) and a higher ear body temperature (36.4 °C vs 36.2 °C, *p* < 0.01) compared to the 136 NHRs without EDIS documentation. No difference in gender (72 % vs 66 % female), age (84 ± 7.5 vs 86 ± 6.4), cognitive status (MMSE 13 ± 8 vs 15 ± 9) or the presence of chronic disease or medication was found.

## Descriptive analysis of the response scale

The NA response rate of the 12 items with graded response alternatives in three levels in the preliminary EDIS varied between 96 and 100 %. The whole range of response alternatives - completely agree/partly agree/do not agree - was used for all of these items. The 13^th^ item concerned temperature, and 60 of the 68 NHRs (88 %) with a suspected first infection event had either an ear or a rectal temperature reported in the study protocol. An examination of the missing data did not reveal any systematic patterns, and the number of missing values was small in the preliminary 13 EDIS items.

### Content validity

The internal content of the preliminary EDIS was explored by an analysis of correlations. The analysis showed that 12 of the 13 preliminary EDIS items (12 statements and one temperature variable) correlated significantly with at least one other statement. The range in number of significant inter-correlations was from 0 (change in “pain”) to 8 (change in “general signs and symptoms of illness”), see Table 2.

**Table 2:** Bivariate correlation between the items in the preliminary Early Detection of Infection Scale (EDIS; twelve statements measuring changes in signs and symptoms and one temperature item

“There is a change in the patient’s…”	Confusion	Aggressiviness	Infirmity/ Apathy	Unrestrained behaviour	Restlessness	Changed appetite	Pain	General signs & symptoms of illness	Expression (of illness) in the eyes	Urinary tract symptoms	Respiratory symptoms	Symptoms of wound infection	Temperature
Confusion^a^	1.00	0.44*	0.00	0.38*	0.48*	0.02	−0.08	−0.31*	−0.25*	0.22	−0.09	0.36*	−0.18
Aggressiveness^a^	0.44*	1.00	−0.01	0.62*	0.45*	−0.06	0.07	−0.29*	−0.27*	0.34*	−0.13	0.20	−0.16
Infirmity/ Apathy^a^	0.00	−0.01	1.00	−0.05	−0.01	0.35*	0.11	0.32*	0.51*	0.04	0.17	0.26*	0.12
Unrestrained behaviour ^a^	0.38*	0.62*	−0,05	1.00	0.44*	−0.12	0.10	−0.25*	−0.29*	0.23	−0.32*	0.15	−0.20
Restlessness^a^	0.48*	0.45*	−0.01	0.44*	1.00	−0.15	0.10	−0.43*	−0.21	0.27*	−0.14	0.22	−0.29*
Changed apetite^a^	0.02	−0.06	0.35*	−0.12	−0.15	1.00	0.15	0.22	0.13	−0.12	0.18	0.19	0.04
Pain^a^	−0.08	0.07	0.11	0.10	0.10	0.15	1.00	0.19	−0.08	0.18	−0.05	−0.05	−0.19
General signs & symptoms of illness^a^	−0.31*	−0.29*	0.32*	−0.25*	−0.43*	0.22	0.19	1.00	0.19	−0.31*	0.44*	−0.09	0.47*
Expression (of illness) in the eyes^a^	−0.25*	−0.27*	0–51*	−0.29*	−0.21	0.13	−0.08	0.19	1.00	−0.05	0.18	0.15	0.12
Urinary tract symptoms^a^	0.22	0.34*	0–04	0.23	0.27*	−0.12	0.18	−0.31*	−0.05	1.00	−0.14	0.24	−0.10
Respiratory symptoms^a^	−0.09	−0.13	0.17	−0.32*	−0.14	0.18	−0.05	0.44*	0.18	−0.14	1.00	0.04	0.34*
Symptoms of nfection^a^	0.36*	0.20	0.26*	0.15	0.22	0.19	−0.05	−0.09	0.15	0.24	0.04	1.00	0.14
Temperature °C	−0.18	−0−16	0.12	−0.20	−0.29*	0–04	−0–19	0.47*	0.12	−0.10	0.34*	0.14	1.00
Number of other statements significantly correlating to respectively statement/temperature	6	6	4	6	6	1	0	8	4	3	3	2	3

*p < 0.05; non-parametric correlation; two-tailed

^a^Response alternatives: Completely agree -Partly agree -Do not agree

### Signs and symptoms in the habitual condition and when NAs suspected infection

In the 68 included patients, the studied signs and symptoms were present in the NHR’s habitual conditions with a range from 18 % (“aggressiveness”) to 62 % (“pain”). When NAs suspected an infection, a change in signs and symptoms (according to the preliminary EDIS) were observed by the NAs ranging from 7 % (“symptoms of wound infection”) to 79 % (“infirmity/apathy”). In the events when the NA suspected an infection and the subsequent evaluation by the researchers resulted in the assessment “no infection”, a change in the studied signs and symptoms was observed by the NAs ranging from 12 % (“symptoms of wound infection”) to 88 % (“infirmity/apathy”). In those events where the following researcher-evaluations instead were “infection”, a change was observed by the NAs ranging from 9 % (“symptoms of wound infection”) to 91 % “infirmity/apathy”), see Table 3.

**Table 3:** Observed signs and symptoms by nursing assistants (NA) in nursing home residents (NHR) in habitual condition (base line; *N* = 68) , when infection suspected by NA (first event of suspected infection; *N* = 68), and when these suspected infections later on were assessed by researchers as “no infection” (*n* = 17), “possible infection” (*n* = 16) or “ infection” (*n* = 35)

Observed signs and symptoms	Present in habitual condition^a^	Change when suspected infection^b^	Change when no infection^b^	Change when possible infection^c^	Change when infection^b^	Wald values^c^
n (%)	68 (100)	68 (100)	17/68 (25)	16/68 (23)	35/68 (51)	
Confusion n (%)	13 (19)	19 (28)	5 (29)	5 (31)	9 (26)	0.23
Aggressiveness n (%)	12 (18)	10 (15)	3 (18)	1 (6)	6 (17)	0.09
Infirmity/ Apathy n (%)	27 (40)	54 (79)	15 (88)	7 (44)	32 (91)	0.38
Unrestrained behaviour (n (%)	15 (22)	12 (18)	3 (18)	3 (19)	6 (17)	0.38
Restlessness n (%)	24 (35)	20 (29)	7 (41)	4 (25)	9 (26)	0.32
Food intake n (%)	22 (32)	32 (47)	11 (65)	5 (31)	16 (46)	0.53
Pain n (%)	42 (62)	21 (31)	6 (35)	6 (38)	9 (26)	0.46
General signs and symptoms of illness n (%) (e.g. fever, hot or cold, shaking, shivering, pale, flushed face)		25 (37)	4 (24)	5 (31)	16 (46)	4.63*
Expression (of illness) in the eyes n (%) (e.g. vacant/hazy/glassy/roaming eyes)		47 (60)	11 (65)	8 (50)	28 (80)	2.04
Urinary tract symptoms n (%)		24 (35)	5 (29)	6 (38)	13 (37)	0.01
Respiratory symptoms n (%)		33 (49)	7 (41)	4 (25)	22 (63)	4.81*
Symptoms of wound infection n (%)		5 (7)	2 (12)	0 (0)	3 (9)	0.08
Temperature °C mean ± SD (range)	36.3 ± 0.5 (34.9 – 37.3)	37.3 ± 1.0 (35.5 – 39.5)	36.7 ± 0.8 (35.5 – 39.1)	37.1 ± 0.6 (35.8 – 38.0)	37.8 ± 0.9 (36.0 – 39.5)	15.29**
Temperature difference °C from baseline mean ± SD (range)		1.1 ± 1.0 (–0.7 – + 3.4)	0.3 ± 0.7 (−0.7 – +2.0)	0.9 ± 0.6 (−0.6 – +1.8)	1.6 ± 1.0 (−0.7 – +3.4)	15.78**

*p < 0.05. **p < 0.01

^a^According to Neuropsychiatric Inventory – Nursing Home version (NPI-NH)

^b^Data from NAs collected via EDIS (Early Detection of Infection Scale); Partly agree and Fully agree has been merged in the analyses

^c^Wald = 4 is approximately equivalent to *p* = 0.05; Wald = 6 is approximately equivalent to *p* = 0.01

The difference in the observed changes in the studied signs and symptoms between events assessed as “infection” and those assessed as “no infection” ranged from 19 % (“food intake”) to 22 % (both “general signs and symptoms of illness” and “respiratory symptoms”).

### Construct validity

In the analysis of the construct validity, individual variables showed that three of the hypothesized signs and symptoms were significantly related to “infection”, namely the items “temperature”, “respiratory symptoms” and “general signs and symptoms of illness” (increased Wald values). These three signs and symptoms arose from NAs observations that “He/She seems to be ill” (Table 3).

### Changes in signs and symptoms and verified infection: analysis of individual variables

Classification of the highly significant developed GLZ model (*p* = 0.000091) was carried out and the percentage of correct classifications of the observed cases was calculated. Of the 59 respondents used in the selected model (i.e. the items “temperature”, “respiratory symptoms” and “general signs and symptoms of illness”), correct alternative responses were predicted in 61 %, with a range from 0 % (six cases assessed by researchers as “possible infection”, none correctly classified) to 84 % (31 cases assessed by the researchers as “infection”, 26 correctly classified). Moreover, 67 % (*n* = 10) of the 15 cases assessed by the researchers as “no infection” were correctly classified. Sensitivity and specificity were calculated for two of the items possible to dichotomize “respiratory symptoms” (51 % respectively 29 %) and “general signs and symptoms of illness” (41 % respectively 30 %).

## Discussion

It is well known that non-specific symptoms and lack of specific ones are common in infections of NHRs [26–28], contributing to delayed diagnosis and treatment [27]. In addition, early signs of infection are very similar to, and also as non-specific as signs of acute illness other than infection [12, 17, 29]. The items in EDIS are therefore similar to the instrument developed by Boockvar et al [29], but in this study we focused on the change from habitual condition related to suspected infection, observed by NAs. Three variables assessed by the NAs in the preliminary EDIS related to verified infection, namely “temperature”, “respiratory symptoms” and “general signs and symptoms of illness”. Although the EDIS instrument (according to the developed model) did not have precision in predicting the middle group, i.e. “possible infection”, it correctly predicted patients with “no infection” and “infection” in 67 and 84 % of the cases, respectively. In the clinical practice, sometimes an infection in an elderly NHR with multiple diseases should not be treated with antibiotics because the NHR is experiencing a palliative phase in the disease trajectory. However, such decisions should not be made by NAs. At the stage when NAs contribute to the team’s early handling of NHRs with possible infections, it seems important to avoid the risk of underestimating as well as overestimating, a patient’s risk of having an infection, so the RN and/or the GP also assess the patient, and initiate, when appropriate, further laboratory tests. This is also of significance for the increasing rate of antibiotic resistance [16]. To our knowledge this is the first study to statistically test the evidence of NA observations by using a systematically developed instrument, the Early Detection of Infection Scale (EDIS), for early detection of infection in elderly NHRs.

None of the atypical signs and symptoms of infection in the preliminary EDIS chosen to indicate that the patient “is not as usual” [18] predicted the presence of infection. However, the presence of atypical signs and symptoms of infection was already frequent in the habitual condition in the studied NHRs, ranging from 18 % (“aggressiveness”) to 62 % (“pain”). Although, this is not surprising considering the high prevalence of dementia in the studied population (72 %), the presence of neuropsychiatric symptoms in the habitual condition (in many patients several symptoms at the same time) may have contributed to the difficulty for NAs to identify such changes. The NAs’ statements about changed behaviour/condition in this study are similar to changes in acute conditions in other studies [2–4, 9, 10, 14, 30–33]. Other findings report specific changes, such as lethargy, weakness, decreased appetite, agitation, disorientation, dizziness, falls and delusions to have high predictive values for acute illness in frail elderly [2, 29]. Our study indicates that such non-specific symptoms are strongly related to each other and, although not significantly, also to more specific signs of illness, i.e. “he/she seems to be ill”. None of the items in EDIS, however, appears as especially highly specific in verifying the presence or absence of infection, i.e. they do not solely correlate with infection or have sufficient sensitivity or specificity. On the other hand, the item “general signs and symptoms of illness”, strongly correlated with several other items, such as “confusion”, “aggressiveness”, “infirmity/apathy”, “unrestrained behaviour”, “restlessness”, “urinary tract symptoms”, “respiratory tract symptoms” and “body temperature”. As we do not know if any of these items are more important than others, it is not enough to take action based on solely reported behavioural changes/conditions if not observed in relation to general signs and symptoms of illness. An important observation is that the rating of the change could be present as well as absent in relation to habitual condition, implying that an individual that normally is confused, anxious and restless may become apathetic and infirm, or vice versa. On the other hand, an individual who normally is unrestrained could become more confused, aggressive, anxious and restless. The difficulty is to understand if the changes are due to infection or other acute conditions in frail elderly individuals. In addition to a medical evaluation, one possible course of action may be to perform analysis of C- reactive protein (CRP) directly at the NH, as the CRP level rises rapidly in response to inflammatory stimuli, especially bacterial infections [34]. Another option is procalcitonin, although not yet widely in clinical use, which might have higher sensitivity [35].

As specific symptoms are often lacking [26–28] the presence of fever is often evaluated as a significant symptom of illness and an important reason for taking further action. Body temperature in the EDIS was strongly correlated to verified infection, with a mean difference in increase of 1.6 °C ± 1.0 °C, reaching a mean temperature of 37.8 ± 0.9 °C, which actually is often considered not to indicate fever [14, 30]. This emphasizes the need to base assessment of fever on the increase from baseline body temperature and not on fixed values. Although there are probably individual differences, the results from this study and others indicate that a mean increase difference of at least 1.1 °C from baseline, reported when the NA suspects an infection, should prompt further action [2, 30, 36, 37].

The deeper purpose of this validation of EDIS was to understand the NAs’ observations “he/she is not as usual” and “he/she seems to be ill”. So how should the RN and GP interpret these observations? According to the results, NAs’ observations of changes in temperature, respiration and general signs and symptoms of illness, often expressed by the more general observation “he/she seems to be ill” should be taken seriously. However, the observation “he/she is not as usual” is a challenge to interpret. Obviously, NAs observe changed behaviour/condition, but whether this is related to infection or not needs to be studied in larger samples. Nevertheless, it seems that such observations should also be taken seriously and lead to follow-up. Future research may be to apply the design of Bookvar et.al [17], e. g that NAs daily document behavioral and functional status, according to EDIS, in NHR to develop the instrument.

## Limitations

A limitation of the study was the lack of a golden standard in the assessment of whether or not an event was an infection. This might have attenuated sensitivity and specificity. However, there were two experienced physicians, who independently assessed the events and during this process had access to medical information about the outcome of the event in the form of recordings by NAs, RNs and MDs, as well as laboratory tests that had been collected as part of the study. In addition, the individual method of documenting in the records might have influenced the available information. Only 44 % of the first events of NA-suspected infections were recorded by the NA through filling in the preliminary EDIS. Therefore, this finding may have consequences for the generalizability of the results, i.e. there may be a possible bias in the selected sample. On the other hand, the reason for not using the EDIS might have been work load, effecting compliance to following research protocols. The small number of events also made it impossible to reduce the items by factor analysis. The strength of the study is the effort to validate common expressions from staff who perform individual care of frail elderly. The EDIS instrument could make communication between healthcare staff more systematic and, hence, enhance the possibility of detecting suspected infection early on.

## Conclusion

In conclusion, the validation of EDIS suggests that the observation of change in “temperature”, “respiratory symptoms” and “general signs and symptoms of illness” made by nursing assistants should be taken seriously in early detection of infection in frail elderly. Also the statement “he/she is not as usual” should lead to follow-up. It seems important to further study the EDIS items, in combination with laboratory tests and medical evaluation to increase the quality of care.

## Additional file

Additional file 1:
**The EDIS instrument 150805.** Word document. (DOC 40 kb)
