# Supplementary material for: Nursing assistants: “He seems to be ill” – a reason for nurses to take action: validation of the Early Detection Scale of Infection (EDIS)
Source: BMC Geriatr. 2015 Oct 12;15:122. doi: 10.1186/s12877-015-0114-0 (PMC4603967; doi:10.1186/s12877-015-0114-0)
Supplement: Additional file 1: — The EDIS instrument 150805. Word document. (DOC 40 kb) [file 12877_2015_114_MOESM1_ESM.doc]

Code_____Name:______________________________Date___________Time________

Describe what you consider as different by ticking the boxes and measure the body temperature. It is important that you tick all boxes

| The differences are: | Completely agree | Partly agree | Do not agree |
| --- | --- | --- | --- |
| Discomfort (expression the eyes)(for example: vacant eyes, hazy eyes, glassy eyes, roaming eyes) |  |  |  |
| Unrestrained (for example: uncontrolled talk, in high spirit) |  |  |  |
| Aggressiveness (in talking and in doing) |  |  |  |
| Restlessness (for example: over-excited, messy, do not sleep, anxiety) |  |  |  |
| Confusion (for example: muddled, increased signs of dementia, hallucination) |  |  |  |
| Infirm (tired and feeble) (for example: sleepiness, decreased mobility, apathy, need more help) |  |  |  |
| Decreased eating (for example: do not open mouth, less appetite, does not want to eat or drink) |  |  |  |
| Pain (for example: tenderness, moaning, tense body) |  |  |  |
| General signs and symptoms of illness(for example: fever, hot or cold, shaking, shivering, pale, flushed face) |  |  |  |
| Respiratory symptoms(for example: out of breath, cough, wheezing) |  |  |  |
| Urinary tract symptoms(for example: often goes to toilet, smarting pain, smell, thick urine) |  |  |  |
| Wound infection symptoms(for example: local redness and swelling, pus) |  |  |  |
| **Breath per minute** |  |  |  |
| Body temperature **Left ear _____________^0^C Right ear____________^0^C** **Rectal____________** ^0^C  No body temperature measured because: | | | |

## Other signs or symptoms, describe:________________________________________________

**________________________________________________________________________________**

**I suspect this is an infection:**

Strong suspicion □ Moderate suspicion □ Low suspicion □ No suspicion □

**I will contact the nurse** Yes □ No □
